# Mode of Presentation of Hypertension in a Group of Hypertensive Patients in Erbil

**DOI:** 10.7759/cureus.48724

**Published:** 2023-11-13

**Authors:** Dahen Tariq, Namir G Al-Tawil, Dzhwar Jamal, Midya Mohammed, Shna I Ahmed, Sarah Brzo, Warveen Nawzad, Sava Halgurd

**Affiliations:** 1 College of Medicine, Hawler Medical University, Erbil, IRQ; 2 Community Medicine, Hawler Medical University, Erbil, IRQ

**Keywords:** blood pressure-lowering medication, screening, treatment, diagnosis, symptoms, hypertension

## Abstract

Background

Hypertension is a major public health problem, and it remains the major preventable cause of cardiovascular and all-cause mortality worldwide. Most cases are asymptomatic and discovered incidentally.

Objectives

The objective of this study is to identify (i) the proportion of hypertensive patients diagnosed incidentally and those diagnosed due to symptoms, (ii) the most common symptoms among patients that present with symptoms, (iii) the presentation of acute elevations of blood pressure in hypertensive patients, and (iv) hypertension-related symptoms affect compliance with medications.

Patients and methods

A cross-sectional study was carried out on 386 hypertensive patients in two public health care centres and public places in Erbil, the capital of the Kurdistan Region of Iraq, from December 20, 2021, to February 7, 2022. Data was collected through interviews with the patients using a questionnaire designed by the researchers. A p-value ≤ 0.05 was considered to be statistically significant.

Results

The majority (76.5%) of patients were diagnosed because they got their blood pressure measured due to symptoms. The likelihood of being diagnosed incidentally was not significantly different with age, gender, age at diagnosis, socioeconomic status, or having one or more risk factors. The most common symptoms were headache followed by lightheadedness. Since diagnosis, 87.7% of subjects reported feeling symptoms with elevations in blood pressure, most commonly headaches followed by lightheadedness. Women and individuals belonging to a low socioeconomic status were more likely to report feeling symptoms. Whether or not patients felt symptoms with elevations of blood pressure, it didn’t significantly affect compliance with medications.

Conclusion

The majority of hypertensive patients are diagnosed once they develop symptoms or complications, and a small percentage are diagnosed incidentally. Screening for hypertension in our region may be inadequate. Treatment rates were high. Hypertension-related symptoms didn’t seem to affect compliance with medications.

## Introduction

Hypertension remains the major preventable cause of cardiovascular disease and all-cause death world-wide [1,2]. Although hypertension can produce symptoms, most cases are asymptomatic and are incidentally discovered on blood pressure measurements, such as through screening programs or opportunistic blood pressure measurements [1,3,4]. An alarming proportion of hypertensive subjects (>50%) were unaware they had the condition in a study done in 17 countries [1,2], and a similar figure (53.6%) was reported in a study conducted in Erbil, Iraq [5].

In a 2019 study done by the non-communicable disease (NCD) Risk Factor Collaboration (NCD-RisC), the global age-standardized prevalence of hypertension in adults aged 30-79 years was 32% (95%CI 30-34) in women, and 34% (95%CI 32-37) in men, and the condition was estimated to affect 1.278 billion people [6]. The prevalence of hypertension is highest in lower-income countries, including Middle Eastern countries, and since the health system is weak in these countries, the number of people who are undiagnosed, untreated, and uncontrolled is also higher [5,7]. Saka et al. reported a prevalence of 40.1% in Erbil [5]. Suboptimal blood pressure has been estimated to account for 92 million disability-adjusted life years [8], and 9.4 million deaths a year can be attributed to complications of hypertension worldwide [7].

Treatment can include lifestyle changes, medication, or both [9,10]. Many effective and inexpensive evidence-backed blood pressure-lowering treatments are available that can reduce the risk of the sequelae of hypertension [2,6,8,11]. Hypertension can be detected at the level of primary health care, and screening programs can reduce the morbidity and mortality associated with hypertension [6,12]. Screening can take the form of opportunistic blood pressure measurement at all appropriate primary care visits (2012 guidelines by the Canadian Task Force on Preventive Health Care [13]) or periodic screening programs (The United States Preventive Services Task Force and European Society of Cardiology (ESC)/European Society of Hypertension (ESH) 2018 guidelines [1,10]).

Our aim is to determine the modes in which this condition presents and how this affects disease management. Also, to identify the proportion of hypertensive patients that were diagnosed incidentally and the proportion that were diagnosed due to symptoms, to identify the most common symptoms among the patients that present with symptoms, to determine how acute elevations of blood pressure present in those with diagnosed hypertension, and to assess how hypertension-related symptoms affect compliance with medications.

## Materials and methods

A cross-sectional study was conducted in healthcare institutions of Erbil city, the capital of the Kurdistan Region of Iraq, including Erbil Teaching Hospital (a city hospital) and Layla Qasim Public Health Center (a primary healthcare centre), as well as some other public places. The study was conducted between December 20, 2021, and February 7, 2022. The data was collected between January 8 and January 22, 2022. Before data was collected, the approval of the Research Ethics Committee of Hawler Medical University, Erbil, was obtained (Approval number: 9). Verbal consent was obtained from every participant.

Epi Info™ 7 software (Centers for Disease Control and Prevention, Atlanta, Georgia, United States) was used to calculate the needed sample size. P (prevalence) was set at 0.50, as this gives the largest sample size. The precision level (e) was set at 5% (0.05) and the confidence level at 95%. The calculated sample size was 384, which we rounded up to 385. We added an additional 30 to the target sample size to overcome non-response. Twenty-nine individuals with hypertension refused to participate, thus the non-response rate was 7%. At the end, 386 samples were collected. The inclusion criteria were any person of any age with diagnosed hypertension (by anyone) or taking anti-hypertensive medications. No exclusion criteria were used. The definition for hypertension most often used by physicians in this region is an in-office systolic blood pressure ≥140 mmHg, and/or a diastolic blood pressure ≥ 90 mmHg. The data was collected through convenience sampling.

Data was collected through a questionnaire designed by the researchers and direct interviews with the participants. Prior to data collection, a pilot study was carried out on 30 participants of the same population, and changes were made to the questionnaire where they were deemed necessary. The questionnaire was reviewed and validated by four community medicine specialists from Hawler Medical University's Department of Community Medicine. The questionnaire was composed of two parts: one part was concerned with sociodemographic characteristics (age, gender, height, weight, occupation, educational level, and house and car ownership status) and the other part included questions related to hypertension. A 12-point scoring system was designed by the researchers to classify the participants into different socioeconomic status categories: 4 points were allocated to “Occupation” (4 points for high-rank occupations, 3 for non-manual work, 2 for skilled manual work, 1 for unskilled manual work, and 0 for student, unemployed, or housewife), 5 points were allocated to “Educational level” (5 for college and above, 4 for institute graduate, 3 for secondary school, 2 for primary school, 1 for being able to read and write, and 0 for being illiterate), 2 points were allocated to “House ownership” (2 for full ownership, 1 for partial ownership, and 0 for a rented house or other living arrangements), and 1 was allocated to “Car ownership” (1 for owning a car, and 0 for not owning a car). The total score (12) was divided into three equal categories: 0-4 (low socioeconomic status), 5-8 (middle socioeconomic status), and 9-12 (high socioeconomic status).

Data entry and data analysis were done using IBM SPSS Statistics for Windows, Version 26.0 (Released 2019; IBM Corp., Armonk, New York, United States). Data was presented in the form of frequencies and percentages for categorical data and mean ± SD for numerical data. Independent samples t-test was used to compare the means of two groups. Associations between categorical variables were measured using the Chi-square test, and when the expected counts for more than 20% of the cells in the contingency table were less than 5, Fisher’s exact test was used instead. A p-value ≤ 0.05 was deemed statistically significant.

## Results

There were 386 participants. The mean age ± SD in years was 55.78 ± 12.84, ranging from 14 to 93. The gender distribution was nearly equal, with 52.3% being female and 47.7% being male. These characteristics and socioeconomic characteristics and status are listed in Table 1 and Table 2, respectively. The mean age ± SD at diagnosis with hypertension was 45.43 ± 11.66 years. The mean ± SD duration since the person was diagnosed with hypertension, was 10.34 ± 9.30 years (Tables 1, 2).

**Table 1:** Basic characteristics of the participants.

Variable	Frequency (n)	Percentage (%)
Age Group		
<40	39	10.1
40-49	68	17.6
50-59	119	30.8
60-69	110	28.5
≥70	50	13.0
Gender		
Male	184	47.7
Female	202	52.3
Smoking		
Smoker	73	18.9
Ex-smoker	58	15.0
Nonsmoker	255	66.1
Total	386	100.0

**Table 2:** Socioeconomic characteristics.

Variable	Frequency (n)	Percentage (%)
Occupation		
High-rank occupation	31	8.0
Non-manual worker	74	19.2
Skilled manual worker	54	14.0
Unskilled manual worker	20	5.2
Unemployed or housewife	197	51.0
Student	10	2.6
Education level		
College and above	76	19.7
Institute	35	9.1
Secondary school	68	17.6
Primary school	64	16.6
Read and write	27	7.0
Illiterate	116	30.1
House ownership		
Full ownership	310	80.3
Partial ownership	25	6.5
Rent	50	13.0
Others	1	0.3
Car ownership		
Yes	240	62.2
No	146	37.8
Socioeconomic status		
Low	159	41.2
Middle	131	33.9
High	96	24.9
Total	386	100.0

More than one-third (35.5%) of the patients were diagnosed at the age of 40-49 years, and 28.5% of patients were diagnosed at 50-59 years old (Table 3).

**Table 3:** Age at diagnosis.

Age Group	Frequency (n)	Percentage (%)
<40	95	24.6
40-49	137	35.5
50-59	110	28.5
60-69	39	10.1
≥70	5	1.3
Total	386	100.0

**Figure 1:**
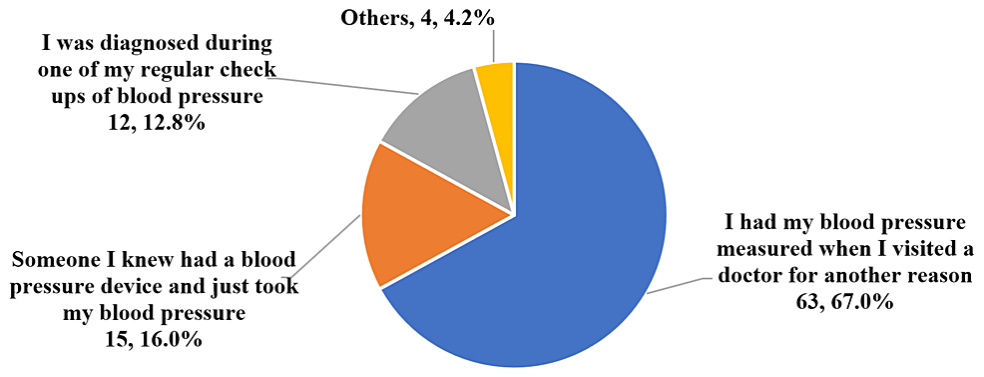
Reason for getting a blood pressure measurement in the participants that didn’t have symptoms.

At the time of diagnosis, 75.6% of the subjects had had their blood pressure measured because they were experiencing symptoms thought to be related to high blood pressure. The other 24.4% didn’t have symptoms. Of these, 12.8% were diagnosed during a regular checkup of blood pressure, and 67.0% had their blood pressure measured when they visited a physician for another reason (Figure 1).

There was no significant association between socioeconomic status (p = 0.231), age (p = 0.855), age at diagnosis (p = 0.908), or gender (p = 0.052) and whether the participant was diagnosed incidentally or due to symptoms (Table 4).

**Table 4:** Association between socioeconomic status and whether the participant was diagnosed incidentally or due to symptoms. * Fisher’s exact test was used. For the rest, Chi-square test was used.

	How they got diagnosed							
	Due to symptoms			Incidentally		Total		p-value
	Frequency (n)		Percentage (%)	Frequency (n)	Percentage (%)	Frequency (n)	Percentage (%)	
Socioeconomic status								
Low	127		79.9	32	20.1	159	100.0	0.231
Middle	97		74.0	34	26.0	131	100.0	
High	68		70.8	28	29.2	96	100.0	
Age								
<40	31		79.5	8	20.5	39	100.0	
40-49	52		76.5	16	23.5	68	100.0	
50-59	87		73.1	32	26.9	119	100.0	0.855
60-69	82		74.5	28	25.5	110	100.0	
≥70	40		80.0	10	20.0	50	100.0	
Age at diagnosis								
<40	73		76.8	22	23.2	95	100.0	
40-49	105		76.6	32	23.4	137	100.0	
50-59	83		75.5	27	24.5	110	100.0	0.908*
60-69	27		69.2	12	30.8	39	100.0	
≥70	4		80.0	1	20.0	5	100.0	
Gender								
Male	131		71.2	53	28.8	184	100.0	0.052
Female	161		79.7	41	20.3	202	100.0	
Total	292		75.6	94	24.4	386	100.0	

**Figure 2:**
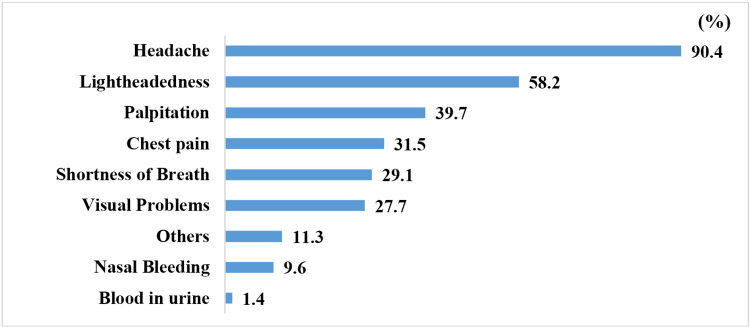
Prevalence of symptoms at the time of diagnosis.

The symptoms reported among the participants that had symptoms are shown in Figure 2, where headache was the commonest symptom (90.4%).

**Figure 3:**
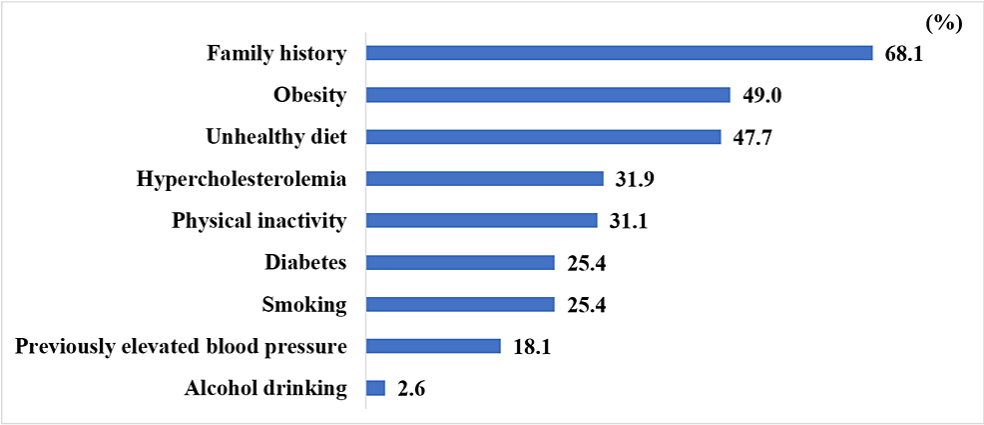
Prevalence of risk factors at the time of diagnosis.

A total of 100 (25.9%) patients developed a complication just before they were diagnosed. The risk factors that the participants had when they were diagnosed are listed in Figure 3. Only nine participants (2.3%) didn’t have any of these risk factors at the time of diagnosis.

No significant association (p = 0.460) was found between having one or more risk factors and whether the patient was diagnosed due to symptoms or incidentally (Table 5).

**Table 5:** Association of having one or more risk factors with whether the participant was diagnosed due to symptoms or incidentally. Fisher’s exact test was used.

	How they got diagnosed							
	Due to symptoms			Incidentally		Total		p-value
	Frequency (n)		Percentage (%)	Frequency (n)	Percentage (%)	Frequency (n)	Percentage (%)	
One or more risk factors								
Yes	286		75.9	91	24.1	377	100.0	0.460
No	6		66.7	3	33.3	9	100.0	
Total	292		75.6	94	24.4	386	100.0	

**Figure 4:**
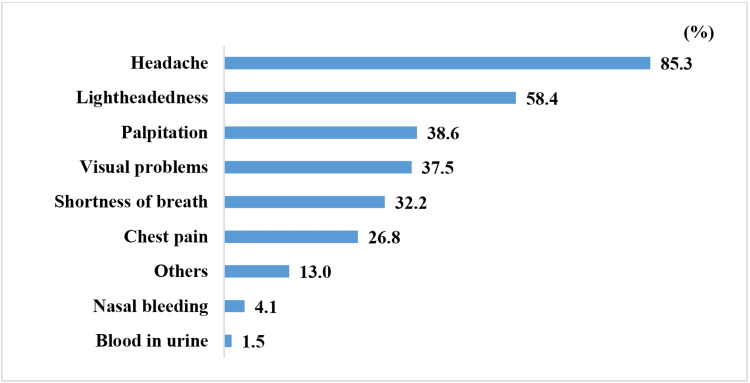
Prevalence of symptoms felt when blood pressure is elevated.

Since they’ve been diagnosed, 87.8% of the participants said they feel symptoms when their blood pressure is high, while 12.2% reported that they didn’t. The symptoms reported among those who said they feel symptoms are listed in Figure 4.

**Figure 5:**
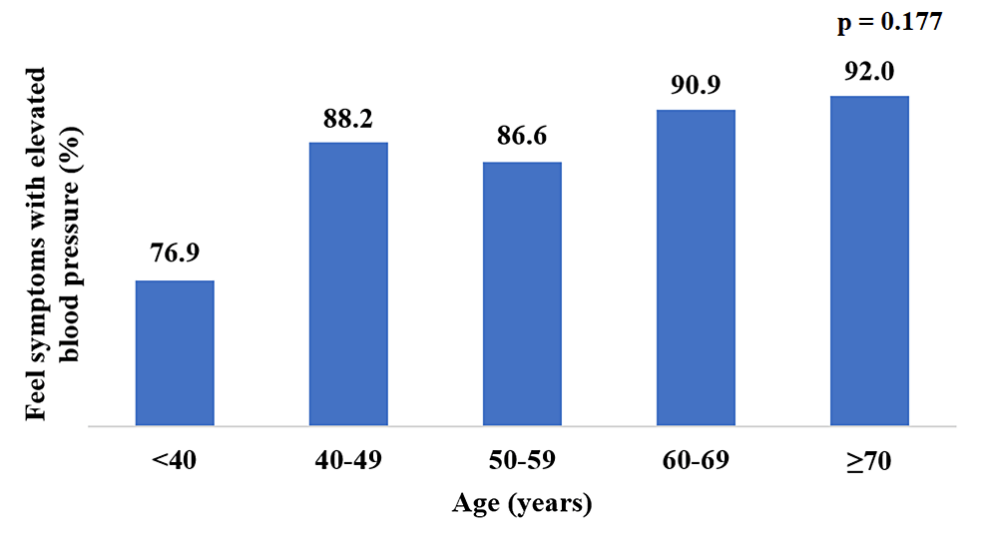
Patients who felt symptoms with elevations in blood pressure and age group. Chi-square test was used.

No significant association (p = 0.177) was found between age and whether or not participants felt symptoms when their blood pressure was elevated (Figure 5). There was a significant association between female gender (p <0.001) and low socioeconomic status (p = 0.002) with feeling symptoms when blood pressure was elevated (Table 6). Results showed also that there was no significant difference (p = 0.138) in the mean duration of diagnosis between the group that reported feeling symptoms and the group that didn’t.

**Table 6:** Feeling symptoms when blood pressure is elevated by age, gender, and socioeconomic status. Chi-square test was used.

	Feel symptoms when blood pressure is elevated							
	Yes			No		Total		p-value
	Frequency (n)		Percentage (%)	Frequency (n)	Percentage (%)	Frequency (n)	Percentage (%)	
Age								
<40	30		76.9	9	23.1	39	100.0	
40-49	60		88.2	8	11.8	68	100.0	
50-59	103		86.6	16	13.4	119	100.0	0.177
60-69	100		90.9	10	9.10	110	100.0	
≥70	46		92.0	4	8.0	50	100.0	
Gender								
Male	149		81.0	35	19.0	184	100.0	<0.001
Female	190		94.1	12	5.9	202	100.0	
Socioeconomic status								
Low	150		94.3	9	5.7	159	100.0	
Middle	106		80.9	25	19.1	131	100.0	0.002
High	83		86.5	13	13.5	96	100.0	
Total	339		87.8	47	12.2	386	100.0	

**Figure 6:**
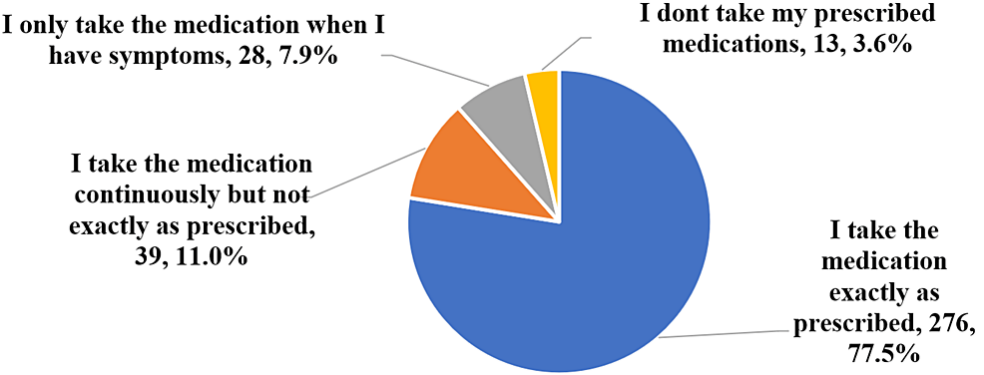
Compliance with medication.

A total of 356 (92.2%) participants were currently prescribed medications, and their compliance with said medications is shown in Figure 6. The participants were divided into those that were taking their medications continuously (88.5%) and those that weren’t (11.5%). There was no significant difference in compliance (p = 0.425) among participants who felt symptoms when their blood pressure was high and those who didn’t (Table 7).

**Table 7:** Association of feeling symptoms when blood pressure is elevated with whether or not the participant takes their medication continuously. Fisher’s exact test was used.

	Taking medication continuously							
	Yes			No		Total		p-value
	Frequency (n)		Percentage (%)	Frequency (n)	Percentage (%)	Frequency (n)	Percentage (%)	
Symptoms with blood pressure								
Yes	282		(89.0)	35	(11.0)	317	(100.0)	0.425
No	33		(84.6)	6	(15.4)	39	(100.0)	
Total	315		(88.5)	41	(11.5)	356	(100.0)	

## Discussion

The presence of symptoms is a trigger for motivating people to seek medical care or adhere to treatments prescribed to them, and an absence of these symptoms in a condition like hypertension could lead to a late diagnosis or even cases going undiagnosed and adversely impact the compliance of patients with medical recommendations. In our society, there’s a stereotype that people don’t seek medical care until they’re sick and feel symptoms, which, if true, would be particularly problematic for asymptomatic conditions like hypertension.

The majority of our participants (75.6%) were diagnosed because they presented with blood pressure-related symptoms, and 25.9% of them were diagnosed after they had developed a complication. Out of those diagnosed incidentally, only 12.8% were diagnosed during a regular checkup of blood pressure. The 2018 ESC/ESH guidelines describe hypertension as an asymptomatic condition that is usually diagnosed incidentally, either through screening programs or opportunistic blood pressure measurements [1]. A study done in Sudan reported a higher, although still not ideal, proportion of individuals being diagnosed during routine clinical check-ups (46%), with the other 54% having been diagnosed after the start of complications (not specified in the study) [14]. A study done in Nigeria reported that the most common age of diagnosis was 40-49 years (46.9%), with 35.8% having been diagnosed before the age of 40 years (7.5% at 20-29 years, 28.3% at 30-39 years) and 17.2% having been diagnosed at or above the age of 50 years [15]. The current study results showed that the age of diagnosis was <40 years in 24.6% of cases, 40-49 years in 35.5% of cases, and ≥50 in 39.9% of cases. Thus, the participants in our study had been diagnosed much later in comparison. Age, gender, and socioeconomic status were not found to affect the likelihood a person was diagnosed incidentally or during screening. Having one or more risk factors for hypertension back when the participant was diagnosed didn’t make a significant difference either.

The two most commonly reported symptoms in the current study were headache and lightheadedness, both at the time of diagnosis (90.4% and 58.2%, respectively) and when there are acute elevations of blood pressure (85.3% and 58.4%, respectively). A study by Granados-Gámez et al. also found these two symptoms to be the most commonly reported among hypertensive patients [16]. In their study, headache was reported by 88% of symptomatic patients, dizziness by 57%, tachycardia by 15%, and flushing by 11%. In the current study, 87.8% of participants reported feeling symptoms when there was an elevation in their blood pressure, while the study by Granados-Gámez et al. reported a rate of 77% [16]. We found that females and those with low socioeconomic status were more likely to report symptoms when blood pressure was elevated. No significant association was seen between age and the years since the patient was diagnosed and the likelihood that the patients felt blood pressure-related symptoms. Granados-Gámez et al. found no significant relationship between beliefs in symptoms and age and gender [16]. They also found that the time elapsed since diagnosis could be an important factor in the formation of beliefs regarding symptoms, which was a conclusion they reached from observing the increasing percentage of patients reporting symptoms as time passed after the diagnosis, and the first year following diagnosis was determined to be a critical time period for the formation of these beliefs. This discrepancy could be due to the fact that the majority (75.6%) of the participants in the current study were initially symptomatic at the time of diagnosis, with a relatively small increase in the percentage that felt symptoms at the time of interview (87.8%), hence the time passed since they were diagnosed did not significantly correlate with whether they reported symptoms with acute elevations of blood pressure.

The majority (92.2%) of participants in the present study were currently on prescribed medication at the time of the study. Globally, 79.7% of women and 77.5% of men with hypertension were on treatment. In Central Asia, the Middle East, and North Africa, 82.8% of women and 80.8% of men with hypertension were on treatment [6]. This difference could be due to the fact that the participants of the current study were visiting public healthcare centres, so it’s more likely they were on medication than the general population. Among participants who were currently on prescribed medications, compliance rates were relatively high; 77.5% of participants were taking their medication exactly as prescribed, and an additional 11% were taking them continuously, even if not exactly as prescribed. Studies have shown compliance rates of 82% in Sudan [14], 61.8% in Ethiopia [17], and 42.2% in Saudi Arabia [18]. The study done by Saka et al. in Erbil showed that 88% of hypertensive individuals were taking their antihypertensive treatment regularly, 5.9% were taking it irregularly, and 6.1% were not taking their medications [5].

A high percentage of hypertensive patients report feeling symptoms and may use these symptoms as a way to estimate their blood pressure and to guide their decisions regarding treatment. The reported percentage of patients who use symptoms to estimate their blood pressure varies from 50-92% [16]. Patients typically interpret these symptoms as a sign that their blood pressure is elevated, and thus an absence of these symptoms is taken as a sign that their blood pressure is controlled, and this has been shown to negatively impact medication compliance. In the study by Granados-Gámez et al, out of 163 hypertensive patients, 55% of those with beliefs about symptoms had failed to adhere to their prescription, while only 25.7% of the patients who didn’t report beliefs about symptoms failed their treatment [16]. The current study found no significant association between participants believing they feel symptoms when their blood pressure is elevated and their compliance with medication. This could be because patients in the area where the present study was done are more likely to be docile and compliant with physician orders, and the physician-patient relationship in our country is of a paternalistic nature usually. Nonetheless, this may be an avenue that’s worth exploring.

Strengths and limitations

To our knowledge, this is the first study done on the mode of presentation of hypertension at the time of diagnosis, and the first study done in Erbil that assesses hypertension-related symptoms and how they affect medication compliance. The size of the sample collected was adequate for the purpose of this study. Interviews were conducted in as uniform a manner as possible, and questions were asked in an open-ended way with minimal or no guidance for the participants when they were asked about what symptoms they felt when they were first diagnosed and when their blood pressure was elevated. However, there’s a potential for recall bias for various data points since they were self-reported. There is a potential for sampling bias as the subjects were patients visiting a healthcare centre/public hospital, making them more health conscious on average, and hence they may be not representative of the whole population. The study was conducted during the coronavirus disease 2019 (COVID-19) pandemic, which may have affected the results as many patients may not have been able to access hospitals or healthcare centres. The study was conducted in only two healthcare institutes in one city, so it may not be reflective of the whole population in this region. The fact that the definition of hypertension can differ from country to country may also make it more difficult to compare prevalence.

## Conclusions

This study revealed that the majority of individuals in the study region are diagnosed with hypertension once they either develop symptoms or complications because of their high blood pressure. The proportion of individuals diagnosed incidentally was small, and the proportion diagnosed through regular check-ups was even smaller. It seems “screening” for hypertension in the region is not adequate. Treatment rates were surprisingly high. It was seen that hypertension-related symptoms didn’t significantly affect compliance with prescribed medications.
